# Resting heart rate (variability) and cognition relationships reveal cognitively healthy individuals with pathological amyloid/tau ratio

**DOI:** 10.3389/fepid.2023.1168847

**Published:** 2023-05-26

**Authors:** Cathleen Molloy, Elizabeth H. Choy, Rebecca J. Arechavala, David Buennagel, Anne Nolty, Mitchell R. Spezzaferri, Caleb Sin, Shant Rising, Jeremy Yu, Abdulhakim Al-Ezzi, Michael T. Kleinman, Robert A. Kloner, Xianghong Arakaki

**Affiliations:** 1 Cognition and Brain Integration Laboratory, Neurosciences, Huntington Medical Research Institutes, Pasadena, CA, United States; 2 Department of Environmental and Occupational Health, University of California, Irvine, Irvine, CA, United States; 3 Clinical Neuroscience Laboratory, Neurosciences, Huntington Medical Research Institutes, Pasadena, CA, United States; 4 Graduate School of Psychology & Marriage and Family Therapy, Fuller Theological Seminary, Pasadena, CA, United States; 5 Department of Psychiatry and Behavioral Sciences, University of Southern California, Los Angeles, CA, United States; 6 Cardiovascular Research, Huntington Medical Research Institutes, Pasadena, CA, United States; 7 Cardiovascular Division, Department of Medicine, Keck School of Medicine at University of Southern California, Los Angeles, CA, United States

**Keywords:** Alzheimer’s disease (AD), alpha event-related desynchronization (alpha ERD), cognitively healthy with normal amyloid/tau ratio (CH-NATs), cognitively healthy with pathological amyloid/tau ratio (CH-PATs), heart rate (HR), vagally mediated heart rate variability (vmHRV), mini-mental state examination (MMSE), root mean square of the successive differences (RMSSD)

## Abstract

**Introduction:**

Resting heart rate (HR) and heart rate variability (HRV) have been linked with cognition in the general population and in older individuals. The knowledge of this aspect of heart-brain relationship is relatively absent in older individuals with early Alzheimer’s disease (AD) pathology. This study explores relationships of the HR, HRV, and cognition in cognitively healthy individuals with pathological amyloid/tau ratio (CH-PATs) in cerebral spinal fluid (CSF) compared to those with normal ratio (CH-NATs).

**Methods:**

We examined therelationshipsbetween1) resting HR and Mini-Mental State Examination (MMSE); 2) resting HR and brain processing during Stroop interference; and 3) resting vagally mediated HRV (vmHRV) and task switching performance.

**Results:**

Our studies showed that compared to CH-NATs, those CH-PATs with higher resting HR presented with lower MMSE, and less brain activation during interference processing. In addition, resting vmHRV was significantly correlated with task switching accuracy in CH-NATs, but not in CH-PATs.

**Discussion:**

Thesethreedifferenttestsindicatedysfunctionalheart-brainconnections in CH-PATs, suggesting a potential cardio-cerebral dysfunctional integration.

## Introduction

### Heart rate and heart rate variability

Heart rate (HR) measures the number of beats per minute (bpm). Heart rate variability (HRV) measures the variation of the inter-beat intervals of successive heartbeats and allows non-invasive evaluation of the autonomic nervous system (1). HRV is usually analyzed in the time domain as the standard deviation of the normal RR-interval (SDNN) and root mean square of the successive differences (RMSSD), as well as in the frequency domain as low frequency (LF), high frequency (HF), and LF/HF ratio. As RMSSD reflects parasympathetic activity, it is also used as vagally mediated HRV (vmHRV) that reflects a psychophysiological index of inhibitory control (2). HRV and HR reflect the balance between acceleratory sympathetic and inhibitory parasympathetic nerve activities and are regulated by the neural structures [e.g., amygdala, hippocampus, prefrontal cortex (PFC)] that are also involved in cognitive and emotional regulation. Therefore, HR or HRV and cognitive performance are related (1, 3–5). The resting HR has been related to cognitive decline in a large cohort study, such that a higher resting HR was related to worse cognitive decline (6). Interestingly, a decelerating HR has been linked with both the selective and global inhibition of motor responses where prefrontal cortical structures are involved, shown in the processing of three variations of a GO-NOGO task (7). In addition, in a study of 104 healthy young participants, resting vmHRV was associated with intraindividual reaction time variability on an attentional task, such that lower resting vmHRV predicted higher variability during the task and better cognitive control (8).

### Risk of early Alzheimer’s disease and HR/HRV

Alzheimer’s disease (AD) remains a considerable challenge for the United States and worldwide. Current treatments for AD are suboptimal and there is an urgent need to identify the risk of early AD before symptoms appear in late-onset AD (9). The pathological changes of AD (amyloid plaques, neurofibrillary tangles as aggregates of tau protein) and synaptic dysfunction precede cognitive impairment in AD by decades (10, 11). This provides a window of opportunity for early detection and intervention when therapies are potentially more effective. Our previous studies have reported that cerebrospinal fluid (CSF) amyloid/tau ratio (2.71) provided 85% sensitivity in identifying AD from cognitively healthy (CH) individuals (12). We use this Aβ_42_/hyperphosphorylated tau ratio cutoff to assign CH participants into two different groups: those with normal (≥2.71: CH-NATs) or pathological (<2.71: CH-PATs) ratio (12). We have reported that compared to CH-NATs, CH-PATs were at higher risk for cognitive decline to mild cognitive impairment (MCI) or AD (12–14). Additionally, CH-PATs presented subtly impaired executive function that implemented in the PFC (12, 15–17). The PFC also regulates HR and HRV (1, 18). However, it is not known if resting HR or HRV are associated with cognition in CH-PATs, such as in a general cognition Mini-Mental State Examination (MMSE), task performance-task switching, or cognitive processing involving inhibition reflected by alpha event-related desynchronization (ERD) (15, 16, 19). Based on the relationships between resting HR, HRV, and these cognitive activities, translational biomarkers, such as non-invasive electroencephalogram (EEG) and electrocardiogram (ECG), can detect synaptic dysfunction and help detect the early risk for cognitive decline. For example, besides the knowledge of the relationships between resting HR or HRV and cognition in young individuals (7, 8), little is known about such relationships in CH-NATS versus CH-PATS. We would like to determine if resting HR or HRV changes might be useful as an early functional biomarker related to subtle cognitive changes of early AD.

Therefore, we explored the relationship between resting HR and MMSE (general cognition) in CH-PATs and compared the findings with CH-NATs or MCI-AD. Further, we considered that CH-NATs, but not CH-PATs, might present heart–brain connections similar to those of the young healthy participants, where a decelerating HR has been linked with both selective and global inhibition of motor responses (GO-NOGO tasks involving the PFC) (7), and lower resting vmHRV has been associated with better executive function and cognitive control (8). Therefore, we hypothesized that in CH-NATs, but not CH-PATs, an accelerating HR is linked with more negative alpha ERD (more brain activation) during an inhibitory control task (Stroop testing) and that greater resting vmHRV is linked with better behavioral performance in task switching.

## Methods

### Participants

The study protocol was approved by the Institutional Review Board (IRB) (HMRI #33797), with signed consent provided by all participants. Participants aged 60 years and above were recruited from advertisements placed in local newspapers and newsletters, the Pasadena Huntington Hospital Senior Health Network, visits to the senior center and assisted living facilities, and word of mouth. The inclusion and exclusion criteria were the same as previously reported (12, 15–17). Briefly, data were collected in line with the Uniform Data Set (20), including demographic data, physical exam, medical history, neuropsychological batteries, and MRI. Neuropsychiatric testing as well as reviews of the clinical data at a consensus conference classified participants as CH, with MCI, or with AD, which are consistent with reported criteria (21–24).

Body fluids, including cerebrospinal fluid (CSF), were collected, with Aß_42_ and total tau measured as reported (12, 15). Briefly, the CSF Aß_42_/t-tau ratio discriminated AD from CH participants with a sensitivity of at least 85% (12). We applied this regression to assign CH participants into two groups: those with a normal CSF Aβ/tau ratio (≥2.7132, CH-NATs) and those with a pathological Aβ_42_/tau ratio (<2.7132, CH-PATs) (12). CH-PATs presented a higher risk for cognitive decline in a longitudinal observation (13). Supplementary Table S1 shows participant characteristics by analysis.

### MMSE and resting HR

The MMSE scores were measured using a standard questionnaire (12). For the resting heart rate, the researcher asked participants to maintain a relaxed sitting position and measured the heart rate using a sphygmomanometer (HEM-790IT; Omeron Healthcare, Inc.) (25, 26). The resting HR before Stroop testing was measured using an ECG during the resting state.

### Stroop, alpha ERD, and resting HR

As a pilot study, the CH individuals went through Stroop or task-switching testing. Cognitive challenge procedures were performed as previously described (15, 17). Briefly, participants were asked to rest with their eyes opened and then their eyes closed for 5 min each while EEG and ECG recordings were taken, followed by cognitive testing. Participants were required to perform one cognitive task per visit to avoid fatigue. For the Stroop, we challenged participants’ interference control by color–word interference testing (i.e., identify the ink color in a colored word), with low load (e.g., the word “Red” printed in red ink, or congruent) and high load (e.g., the word “Red” printed in blue ink, or incongruent) trials (15).

Alpha ERD, a proxy of brain activation during the cognitive task, was analyzed as previously reported (15). Briefly, alpha ERD is the numerical alpha power values that normalized by decibels to the baseline power (before stimulus appear on the screen) (15). The resting HR in this analysis was assessed from a 5 min resting ECG recorded before the Stroop testing.

### Task switching, behavioral performance, and RMSSD

The task-switching paradigm is designed based on a color–word Stroop paradigm. Participants responded to two sequential stimuli (incongruent colored words) in a trial: either a repeat trial (color–color or word–word) or a switch trial (color–word or word–color). ECGs were recorded for 5 min with eyes open before (resting) and during the task switching (17). For this study, only resting ECGs were used for the HRV analysis.

Behavioral performance was assessed using response time (RT) and accuracy (ACC) as previously reported (17). Briefly, response time refers to the time from stimulus onset to correct responses and accuracy refers to the percentage of correctly responded trials over all trials. The resting RMSSD was assessed from a 5 min resting ECG recording (15, 17). Briefly, ECGs were screened and ectopic beats removed, followed by identifying normal RR-intervals (NN) using the Acq-Knowledge software (BIOPAC Systems, Inc), and linear interpolation (Kubios HRV software version 3.2.0), with a subsequent analysis for RMSSD in the time domain.

### Statistical analysis

We used Pearson correlation analysis to estimate the strength of the unadjusted linear relationship between resting HR and MMSE scores in CH individuals (*n* = 57) with CSF amyloid/tau ratios either normal (CH-NATs, *n* = 29) or pathological (CH-PATs, *n* = 28), in participants with MCI (n = 35), and in those with AD (*n* = 30). A one-way ANOVA was performed to compare the HR between the four groups (CH-NATs, CH-PATs, MCI, and AD).

We used Spearman correlation analysis to estimate the strength of the rank correlation between HR and alpha ERD, in CH-NATs (*n* = 13) and in CH-PATs (*n* = 9). We also calculated the Spearman correlation coefficient for resting RMSSD and task-switching accuracy, within CH-NATs (*n* = 18) and within CH-PATs (*n* = 26). Additional statistical information is described in the Supplementary Materials.

In summary, three relationships were explored using a correlation analysis and compared between different cognitive status groups: (1) the relationship between resting HR and general cognition (MMSE); (2) between resting HR and alpha ERD during Stroop processing with low load (congruent) and high load (incongruent) trials; and (3) between resting RMSSD and task-switching performance during low load repeat trials (color–color or word–word) and high load switch trials (color–word or word–color).

## Results

### Resting HR estimated to be moderately related to MMSE in CH-PATs

**FIGURE 1:**
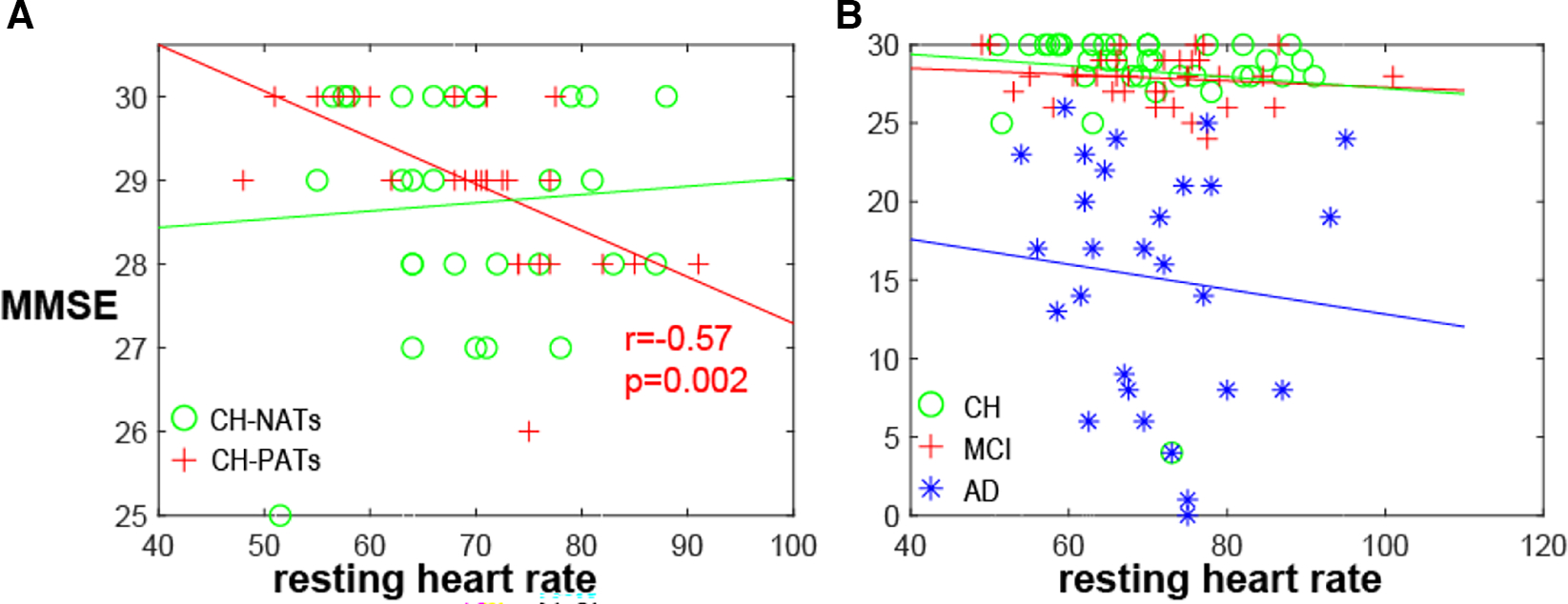
MMSE and resting HR. (A) MMSE negatively correlated with resting heart rate in CH-PATs (*r* = −0.57, *p* = 0.002, red cross), but not in CH-NATs (*r* = 0.07, *p* = 0.71, green circle). (B) Correlation was not significant in all CH (*r* = −0.09, *p* = 0.57, green circle), MCI (*r* = −0.14, *p* = 0.41, red cross), or AD individuals (−0.11, *p* = 0.59, blue star). AD, Alzheimer’s disease; CH, cognitively health; CH-NAT, cognitively healthy individuals with a normal amyloid/tau ratio; CH-PAT, cognitively healthy individuals with a pathological amyloid/tau ratio; HR, heart rate; MCI, mild cognitive impairment; MMSE, mini-mental state exam.

A one-way ANOVA revealed that there was no statistically significant difference in mean HR between CH-NATs, CH-PATs, MCI, and AD (*F*(3,118) = [0.1869], *p* = 0.91). There was a significant negative correlation between resting HR and MMSE among CH-PATs (*r* = −0.57, *p* = 0.002). There was no significant correlation between the same variables in CH-NATs (*r* = 0.07, *p* = 0.71), MCI (*r* = −0.14, *p* = 0.41), or AD (*r* = −0.11, p = 0.59) (Figure 1).

### Resting HR estimated to be positively related to alpha ERD in CH-PATs, but negatively related to alpha ERD in CH-NATs

**FIGURE 2:**
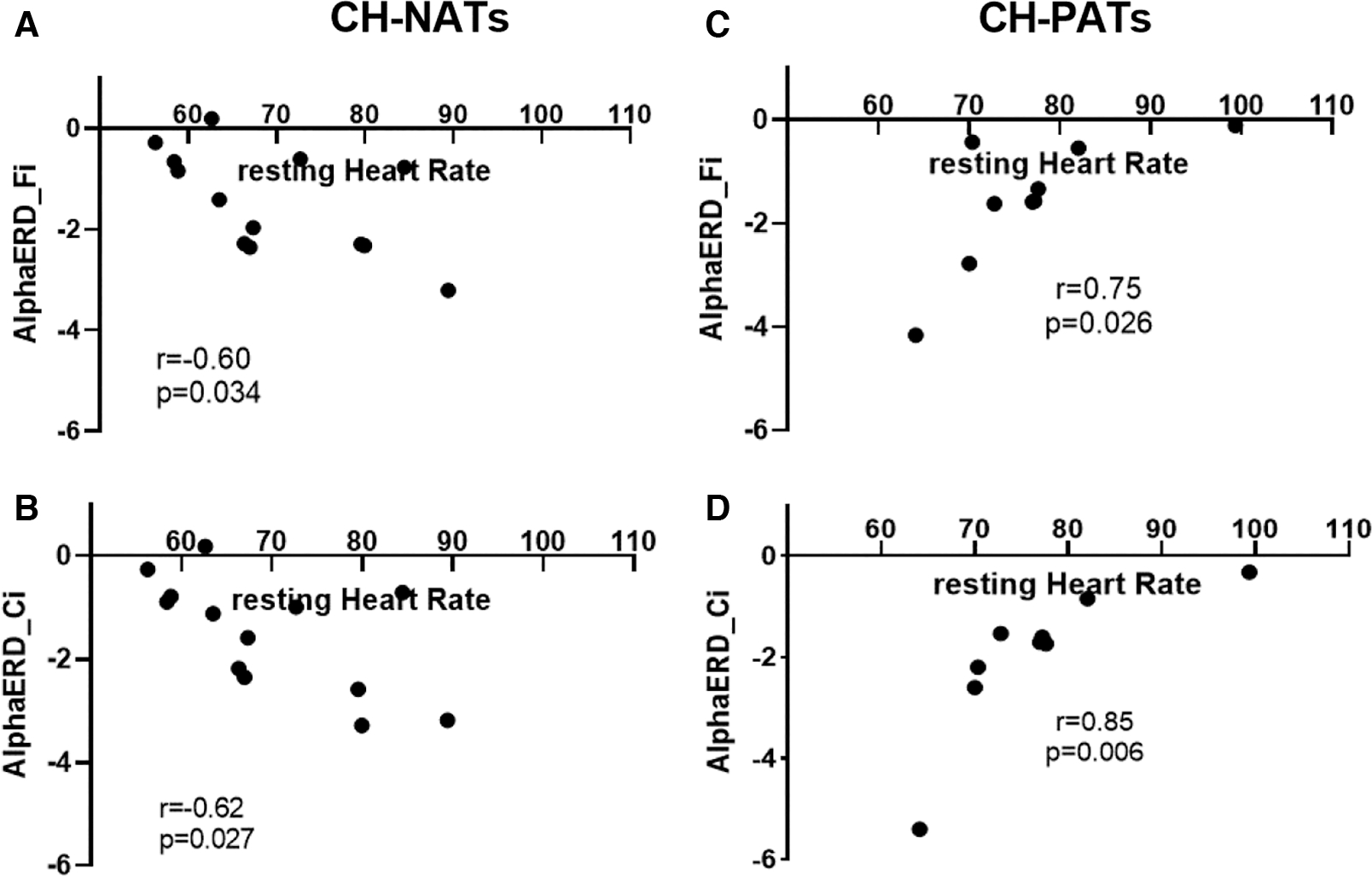
Resting HR and alpha power during Stroop testing incongruent trials. In CH-NATs, resting HR (before the Stroop) negatively correlated with alpha ERD at the frontal region (Fi, A) and at the central region (Ci, B). In CH-PATs, but positively correlated with alpha ERD (Ci) in CH-PATs, resting HR positively correlated with alpha ERD at the frontal region (Fi, C) and at the central region (Ci, D). CH-NAT, cognitively healthy individuals with a normal amyloid/ tau ratio; CH-PAT, cognitively healthy individuals with a pathological amyloid/tau ratio; ERD, event-related desynchronization; HR, heart rate.

For the Stroop task during high load trials, the resting HR was moderately and negatively correlated with alpha ERD in CH-NATs (frontal: *r* = −0.60, *p* = 0.034; central: *r* = −0.62, *p* = 0.027), but highly and positively correlated with alpha ERD in CH-PATs (frontal: *r* = 0.75, *p* = 0.026; central: *r* = 0.85, *p* = 0.006) (Figure 2).

### Resting HRV significantly related to task-switching performance in CH-NATs, but not in CH-PATs

**FIGURE 3:**
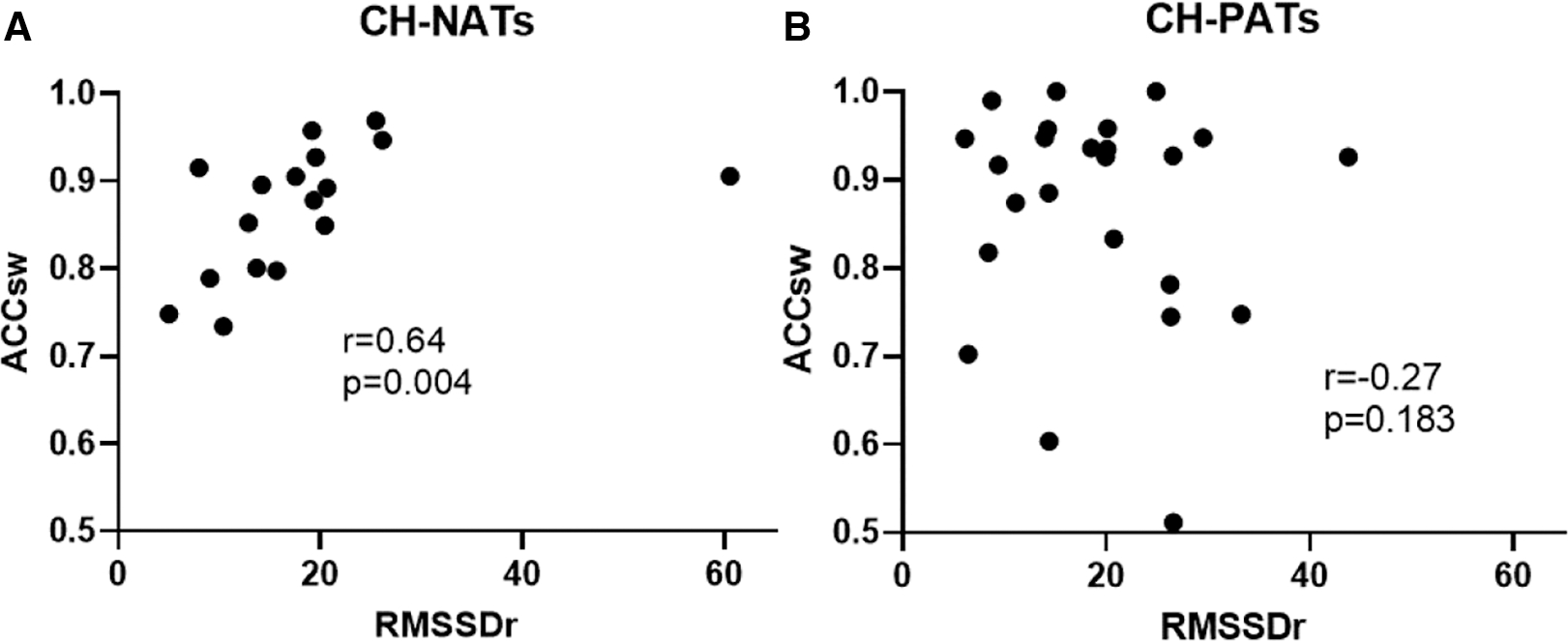
Resting HRV associated with task-switching performance. Resting RMSSD positively correlated with accuracy during switch trials in CH-NATs (A), but not in CH-PATs (B). ACCsw, accuracy during switch trials; CH-NAT, cognitively healthy individuals with a normal amyloid/tau ratio; CH-PAT, cognitively healthy individuals with a pathological amyloid/tau ratio; HRV, heart rate variability; RMSSD, root mean squared of successive differences.

For the task-switching paradigm, the resting RMSSD was moderately and positively correlated with accuracy during switch trials (*r* = 0.64, *p* = 0.004) in CH-NATs, but there was no statistically significant correlation in CH-PATs (*r* = −0.27, *p* = 0.183) (Figure 3).

Additional statistical analyses are described in Supplementary Tables S1, S2.

## Discussion

These results showed that CH-PATs presented unique links between resting HR or HRV and cognitive functions: (1) the resting HR was significantly negatively correlated with MMSE, a finding not observed in the other categories of CH-NATs, MCI, or AD. Specifically, CH-PATs showed a faster HR with worse cognition, while the other groups did not present such a relationship. (2) The resting HR was highly positively and significantly correlated with alpha ERD during Stroop inhibitory control in CH-PATs, while CH-NATs presented sizable negative correlations between resting HR and alpha ERD. In other words, CH-NATs presented an accelerated HR that coincided with greater brain activation (measured as more negative alpha ERD). That is, these individuals were able to speed up the heartbeat to support greater brain activity. However, CH-PATs presented an accelerated HR with less brain activation (less negative alpha ERD). (3) Resting vmHRV was not significantly correlated with the task-switching performance among CH-PATs; while resting vmHRV was significantly correlated with the performance in CH-NATs: more specifically, higher resting vmHRV was moderately but significantly correlated with a higher accuracy when performing the switch trials among CH-NATs. Overall, the heart–brain relationships (between resting HR or HRV and cognitive functions) might help to differentiate CH-PATs from CH-NATs (or MCI-AD), suggesting different potential cerebral–cardiovascular integrations in these two populations. Previous studies have reported associations between resting HR, HRV, and cognition in general populations or older individuals (6, 27); no such relationships were shown in CH-PATs specifically. This analysis helps provide much-needed information that could eventually contribute to non-invasively detecting the risk of early AD using non-invasive, systemic, and translational biomarkers at the CH stage.

### Resting HR and MMSE

We did not observe significant resting HR differences between CH-NATs and CH-PATs, possibly due to their similar age as well as both groups being CH. The sizable and inverse estimated HR-MMSE relationship in CH-PATs may indicate that the cardio-cerebral regulation differed from CH-NATs such that the higher resting HR related to lower MMSE scores (worse cognition). Such a relationship was not observed in CH-NATs, possibly supporting the heart–brain reserve. The observed correlation in CH-PATs is consistent with a study showing that a higher heart rate reserve, or less heart rate acceleration with exercises, was related to less cardiovascular disease and lower all cause mortality (28), where a higher risk of cardiovascular disease was associated with worse cognition (29). The statistically significant HR–MMSE relationship observed in CH-PATs was also not observed in MCI or AD, possibly suggesting individuals with MCI or AD may present worse heart–brain dysregulation than CH-PATs. For example, patients with MCI presented greater sympathetic responses compared to normal cognitive participants (30, 31). Further studies are needed to confirm this relationship, between resting HR and MMSE in CH-PATs, with a larger cohort. This result is in line with a previous large-scale population study from over 20,000 elderly participants using a longitudinal MMSE (approximately 4 years apart). Their resting HR positively related to cognitive decline (MMSE decreased ≥3 points), with an odds ratio of approximately 1.01–1.08 (6). It is possible that their population included both CH-NATs and CH-PATs, which might have blunted the relationship between resting HR and MMSE that was observed in our CH-PAT cohort.

The connection of resting HR and HRV to general health is intriguing. Resting HR has been negatively linked with lifespan across species, such that a lower resting HR is usually associated with a longer lifespan (whales with fewer than 20 bpm and a lifespan over 30 years), and a faster resting HR with a shorter lifespan (rats and mice with over 300 bpm and a lifespan below 5 years) (32, 33). An average heart beats/lifetime of approximately 10^9^ is relatively constant across species, which suggests the heart rate is a marker for metabolic rate (32, 33). A lower heart rate has the benefit of saving metabolic energy and increasing survival. However, a lower heart rate could result in higher central aortic pressure (34). Central aortic pressure, compared to cuff brachial blood pressure, can better predict cardiovascular prognosis and has been associated with end-organ damage (35, 36). Higher central aortic pressure is associated with worse cardiovascular prognosis (37). Higher central blood pressure was related to cognitive decline in participants aged over 50 years (38, 39). Therefore, having a controlled low heart rate without higher central aortic pressure may be beneficial. For example, exercise can help with decreasing HR without compromising central aortic pressure (40, 41).

In humans, a lower resting heart rate has been shown to increase survival in healthy individuals (42), as well as in individuals with coronary artery disease (43). Further, decreasing the heart rate by using pharmaceutical therapy (e.g., beta-blockers) may also increase survival (32). There are increasing interests in the interactions between resting HR or HRV and cognition during pathological conditions because of its easily accessible data. For example, the heart rate increases with cognitive challenge, reflecting brain–heart responses to cognitive load (44). Interestingly, a higher resting HR has resulted in a shortened diastolic phase, more so when the heart rate was <75 bpm (45). This disproportionally decreased diastolic time was related to decreased myocardial oxygen supply and worse survival in healthy individuals and patients with cardiovascular diseases (34). With the confirmative effect on myocardial oxygen supply, the effect of reduced HR on cerebral blood flow is more complicated, depending on physiological and pathological conditions (46, 47). A few mechanisms were proposed, including an association between elevated resting HR and higher shear stress of the vascular wall, adaptive vascular dilation, and increased endothelial reactive oxygen species, followed by greater endothelial damage and increased arterial stiffening—all factors resulting in cardiovascular disease risk and reduced lifespan (34, 48). For example, HR reduction by ivabradine (heart failure treatment) prevented cerebral and renal endothelial dysfunctions in mice with dyslipidemia (34, 49). Dysregulation of perivascular space CSF dynamics from hypertension may also occur with increased HR from angiotensin through actions in the central nervous system (50). Reduced perivascular CSF flow can result in decreased amyloid removal and amyloid burden, especially at the branch points of cerebral blood vessels (51). Our data may reflect an accelerated HR-induced endothelial dysfunction in CH-PATs with lower MMSE scores, or in dysregulation of perivascular space CSF dynamics, which can both result in decreased amyloid removal. Whether those CH-PATs with a higher resting HR will also present a higher risk of cognitive decline or an increased AD pathology burden remains to be determined.

Multiple studies have also supported the connection between lower HRV and reduced cognition in CH populations, including parameters of global cognition, executive functions, and processing speed among others (52). Like cognition, HRV changes with age and gender. HRV analysis via ECG from over 1,500 participants (aged >40 years) has demonstrated lower SDNN with age and BMI, and lower RMSSD with age until 60–69 years, after which RMSSD trended higher (53). Interestingly, women displayed lower SNDD and higher RMSSD than men, and diabetes has been associated with lower HRV in both genders (53). Most participants in this study were female and there were no sizable female–male ratio differences between groups. Our analyses have not looked into male and female comparisons because of the limited sample size. Studies with larger sample sizes are needed in the future to address potential confounding or effect modification by gender.

### Resting HR and alpha ERD during Stroop testing are significantly correlated in CH-PATs

Inhibitory control, or the ability to resist default or dominant responses, is considered to be the common factor of executive functions (54). HR has also been shown to be sensitive to tasks with inhibitory control mechanisms (7). Therefore, we studied the relationship between resting HR and Stroop processing, proxied by alpha ERD (16).

In our study, alpha ERD was estimated to be positively correlated with HR in the CH-PAT group, in response to interference challenges, but not in the CH-NAT group. This significant and positive correlation in CH-PATs in comparison with CH-NATs (negative correlation) might reflect the reduced influence of the PFC on the neurovisceral network, as the parasympathetic activities are regulated by the PFC (1). This finding is also consistent with the frontal dysfunction reported in pre-symptomatic AD (16). The decreased influence of the PFC on CH-PATs may be related to autonomic strain, or to compromised connections between the PFC and amygdala or hypothalamus (1, 3–5). However, the direct link between the frontal cortex and HR regulation in pre-symptomatic AD needs further investigation.

Furthermore, CH-NAT individuals with more negative alpha ERD (increased oxygen demand of the brain) were estimated to be associated with a higher HR (increased effort of the heart), while CH-PAT individuals with more negative alpha ERD (increased oxygen demand of the brain) were associated with a lower HR (decreased effort of the heart). Specifically, CH-NAT individuals who had a higher resting HR presented with more negative alpha ERD, suggesting the HR is increased due to an increased oxygen demand; that is, balanced oxygen demand and oxygen supply from the heart. On the other hand, CH-PATs show the opposite—a lower HR in the setting of more negative alpha ERD (increased oxygen demand), suggesting an autonomic imbalance. For reasons to be determined, CH-PATs might experience an imbalance of oxygen supply and oxygen demand. The two opposite relationships (between resting HR and alpha ERD during Stroop testing) are worthy of further study.

### Resting HR variability significantly associated with task-switching performance in CH-NATs

Resting HRV associated with intraindividual response time variability was studied in a previously published report of 104 healthy young participants. Higher resting vmHRV was associated with lower variability, where lower response time variability was linked with better executive function (8). To our knowledge, there were no similar reports from older individuals. We chose task switching here, as it includes two other core executive function components: working memory and cognitive control (55). Using different age groups (older participants) and paradigms (Stroop-based task-switching paradigm), our study supported a similar relationship in the CH-NAT cohort: specifically, resting RMSSD was significantly associated with the task-switching performance (accuracy). Another slight difference is that the behavioral performance associated with resting RMSSD is accuracy instead of response time. This could be that young individuals differ more in response time, while older individuals differ in accuracy (56). This difference could be attributed to structural and functional changes of an aging-related speed-accuracy trade-off, most likely from aging-related changes in processing and preparing behavioral responses (57).

### HR, HRV, and AD

Both resting HRV and resting HR are regulated by the central autonomic network linking the PFC with cardiac regulation (58). Executive function that implemented in the PFC has been shown to present dysfunction before memory impairment. It has been reported that systemic physiological measures, such as HR or HRV, will be affected by underlying neurodegeneration (27). A summary from the recent literature is shown in Table 1.

Briefly, in participants with an early stage of AD including CH and aMCI when resting HRV (e.g., HF) was lower, a compensatory increase of cortical thickness (in AD signature regions) was reported (65). With further progression, patients with AD presented decreased HRV; however, the HRV change depended on postures (63, 64). Further, HRV-proxied autonomic dysfunction was linked with additional neurodegenerative pathologies, such as white matter lesions (WML) and cerebrovascular burden (61, 62), or the presence of dementia with Lewy bodies (59, 60). Our results added the study of resting HR and HRV association with cognitive functions in the CH individuals who had a pathology of early AD. Therefore, our findings add to the existing knowledge base of the early stage of CH, as well as the relationships between resting HRV, HR, and subtle cognition changes.

### Limitations and strengths

This study has a few limitations, as follows: (1) the sample size is relatively small: the results are exploratory. Conclusions need to be made carefully before results are confirmed in a larger population study. Further female and male differences need to be tested. (2) This is a cross-sectional study. Longitudinal studies are needed to investigate the associations between resting HR, HRV, and cognitive decline. The magnitude and sign of within person correlations can differ from those of group-level correlations (66). A longitudinal study could help with developing a predictive model based on observed correlations within individuals. (3) Hypothetical reasons were raised to explain the unique HR, HRV, and cognition relationships in CH-PATs; but these explanations remain hypothetical and further research is needed to either prove or disprove them. Further studies are needed to elucidate the brain oxygen level, heartbeat diastolic time, and molecular and genetic pathways. (4) Analyses were descriptive; the statistical analysis done was essentially bivariate, with fairly simple models and a small sample. We were intrigued by the possible heart–brain connections and are at the exploratory stage for this analysis. We plan to do a longitudinal study, with a larger sample and more complex statistical models, that can support drawing inferences about a population or predicting early correlates of AD in the preclinical phase.

A major strength of the study is that the resting HR, HRV–cognition relationships in CH-PATs versus CH-NATs were replicated in multiple measures (MMSE, Stroop, tasks switching), and findings were in line with previous reports, in a unique population of CH-PATs. This provides strong albeit limited evidence of potential heart–brain dysfunction in this early AD stage.

### Summary

We have reported that in CH-PATs: (1) a higher resting HR was significantly correlated with lower MMSE, which was not observed in CH-NATs, MCI, or AD; (2) a higher resting HR was significantly correlated with less negative alpha ERD (less brain processing) during Stroop, with opposite findings in CH-NATs; and (3) resting RMSSD was not significantly associated with the task-switching performance, unlike in CH-NATs. These bivariate results support further investigation into whether CH-PATs can be characterized by heart–brain dysfunction. The fact that different tests revealed possible similar heart–brain dysfunction suggests a robust heart–brain dysfunctional phenomenon.

## Supplementary Material

Supplementary Table S2

Supplementary Table S1

## Figures and Tables

**TABLE 1:** Sample studies of ECG/HRV and Alzheimer’s disease and related dementia (ADRD).

Population (age, sample size)	Methods	Results	References
32 participants were MCI-AD, 23 MCI-DLB, and 36 controls were also included. All groups were matched by age (average age = 68–70 years), sex, and education levels	Retrospective design from an existing registry. MCI diagnosis was based on a battery of neuropsychiatric testing, and MCI participants took part in a follow-up appointment in an outpatient unit. ECG recordings were also done and analyzed	The MCI-DLB group had lower levels on SDNN, RMSSD, LF, and HF compared to the MCI-AD group. The MCI-AD group showed no significant differences in HRV compared to the control. LF was shown to be the best diagnostic marker	(59)
80 participants (30 DLB patients, 30 AD patients, and 20 CH controls) were recruited for this study (mean age 79.9 ± 4.7, 79.6 ± 5.6, and 77.2 ± 4.8 years, respectively	5 min recordings were done in a supine position. DLB patients underwent cardiac ^123^I-MIBG scintigraphy in which an injection was given. An MMSE and UPDRS were also administered to evaluate cognitive function	Autonomic dysfunction was greater in the DLB group compared to the AD group. The DLB group had significant decreases in all HRV parameters (except LF/HF ratio) compared to both the AD and control groups, with HF values showing the greatest sensitivity compared to just the AD group	(60)
173 participants (62 aMCI, 73 naMCI, 38 CN) Cross-sectional, age ≥65 years. “Community-dwelling” older adults who had at least one geriatric visit to the Geriatric Outpatient Unit of the local hospital	15 min supine and 10 min upright ECG recordings. CT scan completed to further assess vascular burden along with a 3T-weighted MRI. ApoE genotyping used to test for the presence of ApoE4	Hippocampal atrophy in the aMCI group was higher while WMLs were greater in the naMCI group, both which significantly correlated with cerebrovascular burden. The autonomic response to orthostatic changes (sitting to standing) was distinct between aMCI and naMCI groups	(61)
82 patients with MCI (n = 82), white matter lesions (WML) and no WML (mean age 67.8 + 7.6 and 72.5 + 8.8 years, respectively)	24-hour ECG recordings, MRI, MMSE, Depression BSI	Vascular diseases and autonomic dysfunction (indexed as RMSSD and LF) were seen more in MCI with WML than without WML	(62)
12 patients with probable AD were recruited as the experimental group (5 men, 7 women; age range 55–73 years). Ten cognitively normal participants (4 men, 6 women, age range 55–70 years) were used for the control	Two 15 min ECG recordings—one at rest and another during a “tilt test.” AD patients received oral administration of a cholinesterase inhibitor (eptastigmine)	Power spectrum components were lower in AD patients compared to controls at rest. During the tilt test, there were no changes in the LF or HF components in AD patients while controls experienced an increase in the LF components and a decrease in the HF component. This was only seen in patients who underwent the cholinesterase inhibitor treatment during the tilt test	(63)
20 patients with probable AD (mean age 71 ± 8.2 years) and 7 participants for the control group (mean age 65 ± 2.4 years) were included in this study. No indication of depressive episodes	MMSE was used to indicate severity of dementia. Two 5–10 min ECG recordings—supine and upright. Consecutive R-R intervals and a power spectrum analysis were gathered	AD patients exhibited higher values in the LF parameter compared to controls. The LF/HF ratio showed high specificity in labeling AD patients from controls when upright although the difference in the supine position was not significant	(64)
38 participants (20 HC, 18 aMCI). Participants were recruited from a memory clinic with or without a clinical diagnosis of MCI, and were aged ≥60 years, community-dwelling, and spoke English	Two visits—first a health screening interview and administration of MoCA and Rey’s Auditory Verbal Learning Test. Second, a 10 min ECG recording followed by a 30 min fMRI and another 10 min ECG recording during a Stroop color task and a dual 1-back task	Higher resting HF-HRV or strong HF suppression was related to lower cortical thickness in AD signature regions, supporting a compensatory mechanism in early stage	(65)

AD, Alzheimer’s disease; CH, cognitively healthy; CN, cognitively normal; DLB, dementia with Lewy bodies; ECG, electrocardiogram; HF, high frequency; HR, heart rate; HRV, heart rate variability; LF, low frequency; MCI, mild cognitive impairment; aMCI, amnestic MCI; naMCI, non-amnestic MCI; MMSE, Mini-Mental State Examination; RMSSD, root mean square of the successive differences; SDNN, standard deviation of the normal RR-interval.

## Data Availability

The original contributions presented in the study are included in the article/Supplementary Material, further inquiries can be directed to the corresponding author.

## Abbreviations

AD: Alzheimer’s disease
alpha ERD: alpha event-related desynchronization
CH-NATs: cognitively healthy with normal amyloid/tau ratio
CH-PATs: cognitively healthy with pathological amyloid/tau ratio
CN: cognitively normal
CSF: cerebral spinal fluid
DLB: dementia with Lewy bodies
ECG: electrocardiogram
EEG: electroencephalogram
HF: high frequency
HR: heart rate
HRV: heart rate variability
LF: low frequency
MCI: mild cognitive impairment
aMCI: amnestic MCI
naMCI: non-amnestic MCI
MMSE: Mini-Mental State Examination
PFC: prefrontal cortex
PSD: power spectrum analysis
RMSSD: root mean square of the successive differences
SDNN: standard deviation of the normal RR-interval
vmHRV: vagally mediated HRV
