# Supplementary material for: Resting heart rate (variability) and cognition relationships reveal cognitively healthy individuals with pathological amyloid/tau ratio
Source: Front Epidemiol. 2023 May 26;3:1168847. doi: 10.3389/fepid.2023.1168847 (PMC10428767; doi:10.3389/fepid.2023.1168847)
Supplement: Supplementary file 1 [file Table1.doc]

**Supplementary materials:**

*Statistical analysis:*

Visual assessment of scatter plots suggested the presence of a curved but monotonic relationship between resting HR and alpha ERD (at the central region or at the frontal region) during Stroop testing (incongruent trials), within CH-NATs and within CH-PATS. Similarly, there appeared to be a monotonic but possibly nonlinear relationship between resting RMSSD and task switching accuracy, among CH-NATs. Consequently, we used Spearman correlation analysis to estimate the strength of the rank correlation between HR and alpha ERD, in CH-NATs (n=13) and in CH-PATs (n=9). We also calculated the Spearman correlation coefficient for resting RMSSD and task switching accuracy, within CH-NATs (n=18) and within CH-PATs (n=26).

For assessing whether estimated correlation coefficients differed significantly from zero, P values were calculated using a t-distribution. Correlation coefficients of size .30 or smaller (that is, coefficients between -0.30 and 0.30) were considered to be negligible; coefficients between .30 and .50 in size were considered low; coefficients between .50 and .70 in size, moderate; and coefficients larger than .70, high. The Fisher z-test statistic was used to compare pairs of groups (such as CH-NATS and CH-PATs) and calculate two-tailed P values in order to say whether two correlation coefficients were significantly different. A significance level of .05 was used in interpreting P values.

*Results:*

Characteristics of participants included in the MMSE analysis were described (supplementary **Table S1a**). In terms of correlation between resting HR and MMSE, there was a significant difference between CH-PATs and CH-NATs (Fisher z-test p=.010), but not between CH-PATs and the MCI group (p=.058), nor between CH-PAT and AD (p=.058) (supplementary **Table S2**).

Characteristics of participants included in this Stroop analysis were described (supplementary **Table S1b**). The calculated central-region alpha ERD correlation coefficients differed significantly between CH-NATs and CH-PATS (p<.001), as did the frontal-region alpha ERD correlation coefficients (p=.001) (supplementary **Table S2**).

Characteristics of participants included in the task switching analysis were described (supplementary **Table S1c**). The correlation coefficients for CH-NATs and CH-PATs differed significantly (p=.002) (supplementary **Table S2**).

*Discussion:*

The P value for the resting HR–MMSE correlation in CH-PATs was low enough that it could have withstood conservative correction for multiple testing though the differences in correlation coefficients between CH-PAT and CH-NAT, MCI, or AD, were considerably less significant or non-significant. During Stroop testing, the P values, .006 (central region) and .026 (frontal), for the resting HR–alpha ERD correlations in CH-PATs would not have withstood the Holm–Bonferroni method for multiple-testing correction, but the differences with CH-NATs would have remained statistically significant after multiple-testing correction. During task switching testing, though the P value for CH-NATs, .004, is only borderline significant after being corrected to .048 with the Hold–Bonferroni procedure, the difference with CH-PATs would have remained statistically significant after adjusting for multiple testing.

Some P values (such as those for correlations between resting HR and alpha ERD in CH-NATs) were less than .05, but were higher than .01, for example, and thus of debatable significance given that correction of P values was not done to address multiple testing. Some statements of statistical significance needed to be qualified in light of the fact that P values higher than .004 would not have survived the Holm method for P value-adjustment in this study. A less conservative method, such as the Benjamini–Hochberg procedure (controlling the false discovery rate, rather than the familywise error rate), might be appropriate if the dependence structure of the P values in this study is carefully considered.

For longitudinal study, a linear mixed-effects model could be used with repeated measurements over time on the same individuals to clarify the heart rate–cognition relationships within a given group such as CH-PATs, and also to compare CH-NAT and CH-PAT among individuals whose CSF amyloid/tau ratio might have changed considerably over time. Without multiple measurements on the same individual, there is a risk of confusing the correlations reported in this study (correlations between variables measured on individuals only once each) with correlations within an individual and presuming that a certain heart rate–cognition pattern within an individual indicates pathological amyloid/tau ratio. The magnitude and sign of within-person correlations can differ from those of group-level correlations(Fisher et al., 2018). A longitudinal study could help with developing a predictive model based on observed correlations within individuals.

**Supplementary Tables S1a-1c:**

| **Table S1a.** Characteristics of participants in MMSE analysis. | | | | | | | | | | | |
| --- | --- | --- | --- | --- | --- | --- | --- | --- | --- | --- | --- |
|  | **CH-NATs** | | | **CH-PATs** | **CH** | | | **MCI** | | | **AD** |
| **n** | 29 | | | 28 | 57 | | | 35 | | | 30 |
| **Age (SD)** | 76.7 (7.5) | | | 78.1 (6.0) | 77.6 (6.9) | | | 77.1 (6.3) | | | 77.0 (10.5) |
| **Sex (F/M)** | 18/11 | | | 17/11 | 35/22 | | | 20/15 | | | 17/13 |
| **education** | 16.8 (2.1) | | | 16.5 (2.3) | 16.6 (2.2) | | | 15.9 (2.7) | | | 14.3 (2.5) |
| **resting HR** | 69.3 (9.6) | | | 69.8 (9.9) | 69.6 (9.5) | | | 70.6 (10.9) | | | 70.2 (10.0) |
| **MMSE** | 28.7 (1.3) | | | 29.0 (1.0) | 28.9 (1.1) | | | 27.9 (1.5)^***^ | | | 15.2 (7.7)^***^ |
| ^***^ p<0.001 vs. CH. MMSE: mini–mental state examination; SD: standard deviation. | | | | | | | | | | | |
| **Table S1b.** Characteristics of participants in Stroop testing analysis. | | | | | | | | |  | | |
|  | | **CH-NATs** | | | | | **CH-PATs** | |  | | |
| **n** | | 13 | | | | | 9 | |  | | |
| **Age (SD)** | | 73.8 (9.0) | | | | | 71.8 (7.0) | |  | | |
| **Sex (F/M)** | | 9/4 | | | | | 9/0 | |  | | |
| **education** | | 15.6 (2.5) | | | | | 15.3 (2.0) | |  | | |
| **resting HR** | | 69.7 (10.6) | | | | | 76.7 (10.0) | |  | | |
| **AlphaERD_Fi** | | -1.5 (1.0) | | | | | -1.6 (1.3) | |  | | |
| **AlphaERD_Ci** | | -1.5 (1.1) | | | | | -2.0 (1.4) | |  | | |
| AlphaERD: alpha event-related desynchronization; Ci: central power during incongruent trials; Fi: frontal power during incongruent trials; SD: standard deviation. | | | | | | | | |  | | |
| **Table S1c.** Characteristics of participants in task switching testing analysis. | | | | | | | | | |  | |
|  | | | **CH-NATs** | | | **CH-PATs** | | | |  | |
| **n** | | | 18 | | | 26 | | | |  | |
| **Age (SD)** | | | 77.4 (8.0) | | | 74.0 (8.4) | | | |  | |
| **Sex (F/M)** | | | 13/5 | | | 21/5 | | | |  | |
| **education** | | | 16.0 (2.1) | | | 17.1 (2.4) | | | |  | |
| **resting RMSSD** | | | 18.1 (12.1) | | | 24.6 (21.7) | | | |  | |
| **ACCsw (%)** | | | 83.9 (14.3) | | | 85.2 (13.9) | | | |  | |
| ACCsw: accuracy during switch trials; RMSSD: root mean square of successive differences between normal heartbeats; SD: standard deviation. | | | | | | | | | |  | |

**Supplementary Table S2.** Heart rate–cognition estimated correlations

| **Variables / Group** | **Sample Size** | **Estimated Correlation Coefficient** | **P value** |
| --- | --- | --- | --- |
| **Resting HR, MMSE** |  |  |  |
| **CH-NAT** | 29 | r = 0.07 | .710 |
| **CH-PAT** | 28 | r = -0.57 | .002 |
| **MCI** | 35 | r = -0.14 | .412 |
| **AD** | 28 | r = -0.11 | .590 |
| **CH-PAT v. CH-NAT** | — | — | .010 |
| **CH-PAT v. MCI** | — | — | .058 |
| **CH-PAT v. AD** | — | — | .058 |
| **Resting HR, alpha ERD at central region** |  |  |  |
| **CH-NAT** | 13 | ρ = -0.62 | .027 |
| **CH-PAT** | 9 | ρ = 0.85 | .006 |
| **CH-NAT v. CH-PAT** | — | — | <.001 |
| **Resting HR, alpha ERD at frontal region** |  |  |  |
| **CH-NAT** | 13 | ρ = -0.60 | .034 |
| **CH-PAT** | 9 | ρ = 0.75 | .026 |
| **CH-NAT v. CH-PAT** | — | — | .001 |
| **Resting RMSSD, ACCsw** |  |  |  |
| **CH-NAT** | 18 | ρ = 0.64 | .004 |
| **CH-PAT** | 26 | ρ = -0.27 | .183 |
| **CH-NAT v. CH-PAT** | — | — | .002 |

P values shown were not adjusted for multiple testing.
