# Supplementary material for: Resting heart rate (variability) and cognition relationships reveal cognitively healthy individuals with pathological amyloid/tau ratio
Source: Front Epidemiol. 2023 May 26;3:1168847. doi: 10.3389/fepid.2023.1168847 (PMC10428767; doi:10.3389/fepid.2023.1168847)
Supplement: Supplementary file 2 [file Table2.doc]

Supplementary materials:

**Supplementary Table S2.** Heart rate–cognition estimated correlations.

| **Variables / Group** | **Sample Size** | **Estimated Correlation Coefficient** | **P value** |
| --- | --- | --- | --- |
| **Resting HR, MMSE** |  |  |  |
| **CH-NAT** | 29 | r = 0.07 | .710 |
| **CH-PAT** | 28 | r = -0.57 | .002 |
| **MCI** | 35 | r = -0.14 | .412 |
| **AD** | 28 | r = -0.11 | .590 |
| **CH-PAT v. CH-NAT** | — | — | .010 |
| **CH-PAT v. MCI** | — | — | .058 |
| **CH-PAT v. AD** | — | — | .058 |
| **Resting HR, alpha ERD at central region** |  |  |  |
| **CH-NAT** | 13 | ρ = -0.62 | .027 |
| **CH-PAT** | 9 | ρ = 0.85 | .006 |
| **CH-NAT v. CH-PAT** | — | — | <.001 |
| **Resting HR, alpha ERD at frontal region** |  |  |  |
| **CH-NAT** | 13 | ρ = -0.60 | .034 |
| **CH-PAT** | 9 | ρ = 0.75 | .026 |
| **CH-NAT v. CH-PAT** | — | — | .001 |
| **Resting RMSSD, ACCsw** |  |  |  |
| **CH-NAT** | 18 | ρ = 0.64 | .004 |
| **CH-PAT** | 26 | ρ = -0.27 | .183 |
| **CH-NAT v. CH-PAT** | — | — | .002 |

P values shown were not adjusted for multiple testing.
